# The effect of probability discounting on reward seeking: a three-dimensional perspective

**DOI:** 10.3389/fnbeh.2014.00284

**Published:** 2014-08-25

**Authors:** Yannick-André Breton, Kent Conover, Peter Shizgal

**Affiliations:** ^1^Department of Neuroscience, University of Minnesota, Twin CitiesMinneapolis, MN, USA; ^2^Groupe de Recherche en Neurobiologie Comportementale, Department of Psychology, Center for Studies in Behavioural Neurobiology, Concordia UniversityMontreal, QC, Canada

**Keywords:** brain-stimulation reward, decision-making, operant conditioning, risk, subjective probability, subjective value

## Abstract

Rats will work for electrical stimulation of the medial forebrain bundle. The rewarding effect arises from the volleys of action potentials fired by the stimulation and subsequent spatio-temporal integration of their post-synpatic impact. The proportion of time allocated to self-stimulation depends on the intensity of the rewarding effect as well as on other key determinants of decision-making, such as subjective opportunity costs and reward probability. We have proposed that a 3D model relating time allocation to the intensity and cost of reward can distinguish manipulations acting prior to the output of the spatio-temporal integrator from those acting at or beyond it. Here, we test this proposition by varying reward probability, a variable that influences the computation of payoff in the 3D model downstream from the output of the integrator. On riskless trials, reward was delivered on every occasion that the rat held down the lever for a cumulative duration called the “price,” whereas on risky trials, reward was delivered with probability 0.75 or 0.50. According to the model, the 3D structure relating time allocation to reward intensity and price is shifted leftward along the price axis by reductions in reward probability; the magnitude of the shift estimates the change in subjective probability. The predictions were borne out: reducing reward probability shifted the 3D structure systematically along the price axis while producing only small, inconsistent displacements along the pulse-frequency axis. The results confirm that the model can accurately distinguish manipulations acting at or beyond the spatio-temporal integrator and strengthen the conclusions of previous studies showing similar shifts following dopaminergic manipulations. Subjective and objective reward probabilities appeared indistinguishable over the range of 0.5 ≤ *p* ≤ 1.0.

## 1. Introduction

To forage successfully, an animal must trade off potential benefits, costs, and risks. Each of these factors is multidimensional. For example, benefits may be arrayed in terms of their kind (e.g., food, water, nesting material), amount, and quality (e.g., the concentration of nutrients). Costs include both the expenditure of energy to locate, procure, and handle a prey item and the time required to do so. Risks include the uncertainty that a given action will produce its intended result and the likelihood of encountering a predator. An influential account of foraging (Charnov, 1976) implicitly equips the animal with computational machinery that boils down multiple determinants so as to represent each available course of action by a single, continuously updated quantity, expressed in a common currency.

The computational processes involved in foraging include psychophysical, combinatorial, and decisional components. Psychophysical processes translate an objective variable, such as the concentration of sugars in a berry, into a subjective one, such as the intensity of the rewarding effect produced by consumption of that prey item. The “boiling-down” operation combines the subjective estimates of benefits, costs, and risks so as to yield an overall assessment of net payoff, which serves as the currency for the decisions that allocate behavior across available prey items and competing activities.

Operant-conditioning methods have long been used to study foraging behaviors and their neural substrates in simplified laboratory environments where the subject can work to earn rewards by performing tasks such as lever pressing. By substituting rewarding brain stimulation for natural goal objects, such as food or water, the experimenter can control the strength, and timing of reward with precision while achieving very high rates of data collection, under stable physiological conditions. New optogenetic methods (Yizhar et al., 2011) can restrict the stimulation to specific neural populations, such as neurons in a specific brain region that express a particular neurotransmitter. Through application of such methods, neural circuitry subserving the computational processes involved in foraging can be identified, and a mechanistic account of the behavior can be developed.

In the present study, rewarding electrical brain stimulation stands in stead of a prey object, and its strength serves as the proxy for prey quality. The cost variable manipulated is the time required to procure a stimulation train. Such opportunity costs represent rewards forgone: the benefit that would have accrued had the next most valuable course of action (e.g., grooming, resting, or exploring) been pursued. The risk variable explored is the probability that a reward will be delivered once the subject has invested the required work time. (Thus, we use “risk” to mean “probabilistic” throughout the remainder of this paper.) The present study is based on a model of intracranial self-stimulation that describes how the strength, cost, and risk of the electrical reward determine the allocation of behavior toward procuring additional stimulation trains. To test basic assumptions of the model and to obtain information about the psychophysics of risk, the trade-off between the strength and cost of rewarding medial forebrain bundle stimulation was assessed at different levels of reward probability.

### 1.1. Shizgal's reward-mountain model

**Figure 1:**
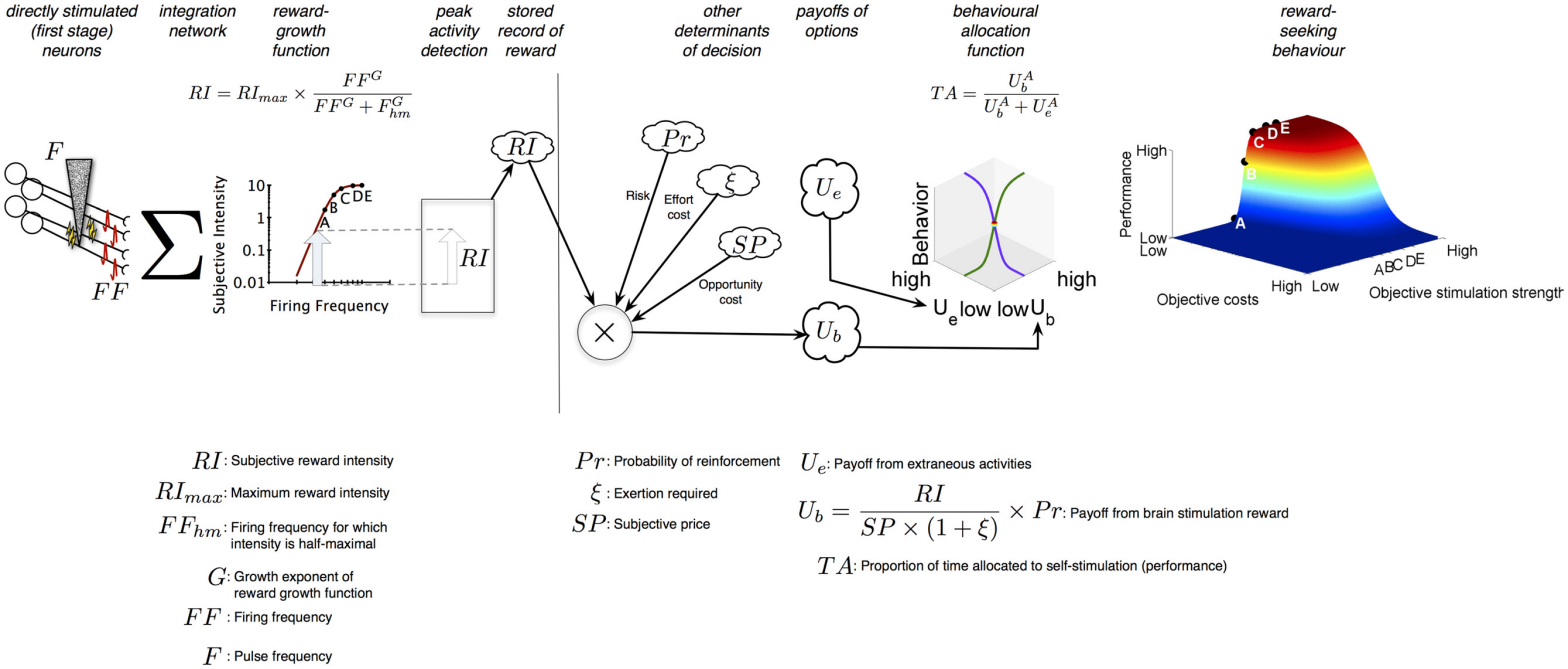
**Sequence of events in the decision to press**. When a rat harvests a brain stimulation reward, the stimulation (*F*) induces a volley of action potentials (*FF*) that travel to a network that integrates the injected signal over space and time (Σ). This integration results in a subjective reward intensity (*RI*); the peak activity of this subjective intensity signal is committed to memory in an engram. In parallel, the probability of reinforcement (*Pr*), the amount of time required (*P*), and the effort invested in acquiring rewards (ξ) is also determined, turned into subjective variables (risk, opportunity cost, and effort cost) and committed to memory. Their scalar combination provides the rat with the payoff it can expect from self-stimulation activities (*U*_*b*_). A comparison of the payoff from self-stimulation with the payoff the rat expects from all other activities it can perform in the operant box (*U*_*e*_) provides the rat with the proportion of time it will spend engaged in self stimulation-related activities (*TA*).

Figure 1 summarizes the Reward-Mountain model. The electrode (shown on the left) triggers a volley of action potentials in excitable neural processes passing close to the tip; some subset of the stimulated neurons gives rise to the rewarding effect. The induced firing frequency (*FF*) in these neurons is determined by the pulse frequency (*F*) according to a logistic function (not shown): as frequency is increased, the induced firing frequency is initially scalar but then rolls off and approaches asymptote as the frequency becomes very high (>300 Hz) (Solomon et al., 2010). The post-synaptic impact of the volley summates temporally and spatially (Σ) so as to implement an aggregate rate code. In such a code, the effect of two pulse trains of equal duration is the same provided that they induce the same total number of firings. Thus, a rat will choose equally between a low-current, high-frequency pulse train that fires a small number of fibers at a high rate and a high-current, low-frequency pulse train that fires a larger number of fibers at a lower rate (Gallistel, 1978; Simmons and Gallistel, 1994). The aggregate rate of firing is translated into a subjective reward intensity (*RI*) by a logistic “reward-growth” function (Gallistel and Leon, 1991; Simmons and Gallistel, 1994; Arvanitogiannis and Shizgal, 2008; Hernandez et al., 2010; Breton et al., 2013), as shown in the 2D graph, and the peak reward intensity generated by the stimulation train is committed to memory (cloud icon) (Gallistel et al., 1974). The location parameter of the reward-growth function is the frequency that produces a reward of half-maximal intensity, *F*_*hm*_.

The operations to the left of the vertical line in Figure 1 transform an objective variable, the induced firing frequency, into a subjective variable, the peak subjective reward intensity. Analogous transformations return *SP*, the subjective value of the work time required to earn a reward (*P*) (Solomon et al., 2007; Breton et al., 2013) (called the “price” of the reward), *P*_*r*_, the subjective probability that the reward will be delivered upon payment of the price, and ξ, the subjective rate of exertion entailed in working for the reward. These subjective values are recorded in memory together with *RI*.

The Reward-Mountain Model follows the precedent of the generalized (Baum and Rachlin, 1969; Killeen, 1972; Miller, 1976) and single-operant (Herrnstein, 1970, 1974) matching laws by combing, in scalar fashion, the stored subjective values pertaining to the reward and to the conditions for obtaining it. Dividing *RI* by *SP* expresses the subjective reward intensity as a rate experienced while working. The expression for the net payoff from work (*U*_*b*_) discounts this rate by the subjective probability of reward receipt and by a quantity related to the rate of subjective exertion during work. Together, the maximum subjective reward intensity, the subjective reward probability, and the subjective rate of exertion entailed in holding down the lever determine the price, *P*_*e*_, at which a maximally intense brain-stimulation reward produces the same net payoff as the “leisure” activities that compete with lever pressing, such as resting, grooming, and exploring.

The final stage of the Reward-Mountain Model determines the proportion of time spent working as a function of the relative payoffs from work (*U*_*b*_) and leisure (*U*_*e*_) activities. Each payoff is raised to an exponent (*A*) that reflects the sensitivity of time allocation to the price of the reward. When the exponent is small, changes in price have only a modest effect, and allocation falls off gradually as price is increased. The higher the value of *A*, the more closely the allocation curves (3D line graph) approximate a step function. When the price equals *P*_*e*_ and the stimulation is maximally rewarding, the rat allocates half of its time to work and half to leisure.

The 3D surface shown at the right of Figure 1 plots time allocation as a function of the objective strength (*F*) and cost (*P*) of the reward. This surface is dubbed the “reward mountain.”

The Reward-Mountain model is the sole quantitative account proposed to date that links induced neural firing to reward pursuit while accommodating the contributions of reward costs and risk and the transformation of objective task variables into subjective values. Two validation studies (Arvanitogiannis and Shizgal, 2008; Breton et al., 2013) provide support for the model, which has been applied successfully in multiple pharmacological experiments (Hernandez et al., 2010, 2012; Trujillo-Pisanty et al., 2011, 2014).

### 1.2. The present study

A key feature of the Reward-Mountain Model is the link it provides between observed changes in behavior resulting from an experimental manipulation and the stage of processing at which that manipulation produces its effect. The vertical line in Figure 1 separates the stages prior to and beyond the output of the reward-growth function. The non-linear form of the reward-growth function makes it possible to distinguish the two sets of changes (Arvanitogiannis and Shizgal, 2008; Hernandez et al., 2010; Breton et al., 2013). Manipulations that act to the left of the vertical line, such as varying the number of directly stimulated neurons, shift the reward mountain along the strength axis but not along the price axis. In contrast, manipulations that act to the right of the vertical line shift the reward mountain along the price axis but not along the strength axis. This latter postulate is tested in the present study. As shown in Figure 1, reward probability is combined with the other subjective attributes of reward and the reward-procuring task to the right of the vertical line, beyond the output of the reward-growth function. This stands to reason: reward-intensity signals must be computed before they can be combined with reward probability. Altering the likelihood that the rat will be paid following satisfaction of the response requirement should not alter how the induced frequency of firing is translated into subjective reward intensity. In contrast, altering the odds that work will result in reward delivery should change the subjective assessment of the net payoff from work. According to the Reward-Mountain Model, the size of the resulting shift along the price axis reflects the rat's subjective assessment of the change in reward probability. Thus, the present study not only tests a key postulate of the Reward-Mountain Model concerning the direction of shifts produced by manipulating reward probability but also sheds light on how objective probabilities are translated into subjective ones. The nature of that transformation plays a crucial role in theories of human decision making (Kahneman and Tversky, 1979; Prelec, 1998) but has not been investigated as extensively in laboratory animals (Kalenscher and van Wingerden, 2011).

To attain the dual objectives of the study, the reward mountain was measured under two randomly interspersed conditions: riskless trials on which reward was always delivered every time the response requirement had been met and risky trials on which satisfaction of the response requirement was rewarded probabilistically.

## 2. Materials and methods

### 2.1. Surgical procedure

All procedures were carried out in accordance with the requirements of the Canadian Council on Animal Care and with the approval of the Concordia University Animal Research Ethics Committee. A total of 10 rats were used in the experiment. All rats were handled daily for 1 week prior to macroelectrode implantation.

Bilateral macro-electrodes were aimed stereotaxically at the lateral hypothalamic level of the medial forebrain bundle of Long-Evans rats (Charles River, St-Constant, QC) weighing at least 350 g at the time of surgery. The electrodes were fashioned from 00-gauge insect pins insulated to within 0.5 mm of the tip with Formvar enamel. Rats received a subcutaneous injection of Atropine (0.02–0.05 mg/kg) to reduce mucous secretions, an intra-peritoneal injection of a Ketamine/Xylazine cocktail (87 mg/kg and 13 mg/kg, respectively) to induce anaesthesia, subcutaneous buprenorphine (0.05 mg/kg) as an analgesic, and intramuscular Penicillin (0.3 ml) to reduce infection. Rats were maintained on 0.5% isofluorane at a flow rate of 800 ml/min for the duration of stereotaxic surgery. Stereotaxic coordinates for stimulating electrodes were 2.8 mm posterior to bregma, 1.7 mm lateral to the midline, and halfway between 9 mm from the skull surface and 8.2 mm from dura mater. A return wire was affixed to two of the skull screws anchoring the implant, and an Amphenol connector was soldered to the free end. The unsharpened end of each electrode was soldered to a copper wire, which, in turn, was attached to a gold-plated Amphenol connector. The Amphenol connectors were inserted into a McIntyre miniature connector (Scientific Technology Centre, Carleton University, Ottawa, ON, Canada), which was attached to the skull and skull-screw anchors with dental acrylic.

Immediately following surgery, rats were given a second injection of buprenorphine (0.025 mg/kg). They were also given mild analgesic injections (Anafen, 5 mg/kg) 24 and 48 h after surgery. Rats were allowed to recover for at least 1 week from the day of surgery before screening for self-stimulation began.

### 2.2. Behavioral protocol

Following surgical implantation of stimulation electrodes, rats were screened for self-stimulation behavior in manually operated operant chambers in which every depression of the lever triggered a 500 ms train of 0.1 ms cathodal pulses delivered to one of the hemispheres, on a continuous reinforcement schedule. Only animals who quickly learned to avidly depress the lever without stimulation-induced involuntary movements or evident signs of aversion (vocalizations, withdrawal or escape behaviors) were retained for this study. Currents ranging from 200 to 1000 μA and pulse frequencies from 50 to 200 Hz were tested and adjusted for each rat and electrode to provide optimal performance.

After screening, rats underwent operant training in the computer-controlled testing boxes that would eventually be used for the experiment. All tests were conducted in the dark phase of their light/dark cycle. The operant task was to hold down a lever so as to accumulate time on a clock that triggered reward delivery (either reliably or probabilistically) once a criterion called the “price” was attained (Breton et al., 2009). If the the lever was released before the criterion was reached, the clock paused, and it began accumulating time again when the lever was again held down.

We define a trial as a period of time during which the strength and cost of the rewarding stimulation is constant, and the rat has the opportunity to earn multiple trains (25). Rats were first presented with a repeating sequence of 10 trials in which the first two trials were identical and each subsequent trial delivered stimulation trains that decremented in frequency by 0.1 common logarithmic steps. Trials were signaled by a house light that flashed for the duration of a 10-s inter-trial interval; priming stimulation consisting of the highest frequency the animal could tolerate at a train duration of 500 ms was delivered 2 s before the end of the trial. Each trial lasted 25 times the price, allowing the rat to obtain a maximum of 25 rewards if it held the lever continuously throughout the trial. The price, pulse frequency, and probability of reinforcement were held constant for the duration of a trial. During this first phase of training, the price was set to 1 s. Pulse frequencies were adjusted throughout to span a range of frequencies that produced high time allocation ratios, a range that produced low time allocation ratios, and a range that produced intermediate time allocation ratios.

When performance on such training “frequency sweeps” was reliably high on high-frequency trials and low on low-frequency trials, as determined by visual inspection, rats were presented with a repeating sequence of 10 trials in which the first two trials were identical and each subsequent trial delivered stimulation that incremented in price by 0.125 common logarithmic steps. The frequency delivered on these trials was as high as the animals would tolerate without involuntary stimulation-induced movements or vocalizations. Training on these “price sweeps” was considered complete when low prices produced reliably high time allocation ratios and high prices produced reliably low time allocation ratios, as determined by visual inspection.

Following “price-sweep” training, rats were presented with a repeating sequence of 10 trials in which the first two were identical and each subsequent trial decremented in frequency and incremented in price. The prices and frequencies of stimulation were arrayed along a line that passed through two points: (1) a price of 4 s and the frequency delivered during price sweeps and (2) the price and frequency that produced half-maximal performance on price and frequency sweeps, respectively, in logarithmic space. Training on these “radial sweeps” was considered complete when high-payoff (high frequency, low price) trials produced reliably high time allocation ratios, and low-payoff (low frequency, high price) trials produced reliably low time allocation ratios by visual inspection.

When training was complete, animals progressed to the discounting portion of the experiment. Preliminary fits to the frequency, price, and radial sweeps were used to aim three vectors in the sample space of prices and pulse frequencies: a vector of 9 frequencies obtained at a price of 4 s (the frequency pseudo-sweep), a vector of 9 prices obtained at the highest frequency the animal would tolerate (the price pseudo-sweep), and paired vectors of 9 prices and frequencies, arrayed along the line that passed through the intersection of the frequency and price pseudo-sweeps and through the anticipated value of *F*_*hm*_ and *P*_*e*_. The vectors thus formed describe the set of price-frequency pairs that would be delivered on certain (*Pr* = 1.00) trials. These vectors were shifted leftward along the price axis by 0.125 common logarithmic units (decreasing all prices on those trials by roughly 25%) for the list of price-frequency pairs that would be delivered on risky trials on which the probability of reinforcement following successful completion of the work requirement was 0.75. The vectors were shifted leftward along the price axis by 0.30 common logarithmic units (decreasing all prices on those trials by roughly 50%) for the list of price-frequency pairs that would be delivered on risky trials on which the probability of reinforcement following successful completion of the work requirement was 0.50.

The first probability condition rats encountered was 0.75 (P1vp75). A master list combining the frequency, price, and radial pseudo-sweeps for the *Pr* = 1.00 and *Pr* = 0.75 conditions was assembled. The central 5 price-frequency pairs of each pseudo-sweep (the 3rd through the 8th elements of each pseudo-sweep when ordered by payoff) were repeated in this master list. As a result, we collected twice as many observations of the time allocation ratio in the dynamic range of the performance curve, reducing our uncertainty about the position of the curve along either the frequency or price axes. This master list was then randomized to yield a new list, providing one full “survey,” or a full description of performance at each point in the tested parameter space.

Trials were grouped in repeating triads. Throughout the experiment, fixed values of the price and pulse frequency were in effect on the first and last trials of each triad; in contrast, the price and pulse frequency on the central trial varied from one triad to the next. The first (“leading”) trial of each triad offered a strong reward at a low price. The pulse frequency was set to the highest value the animal could tolerate, and the price was 1 s. The last (“trailing”) trial of each triad offered a worthless reward at an equally low price of 1 s. The pulse frequency on the trailing trials was set to 10 Hz, a value too low to support lever pressing. Data from the leading and trailing trials provide a index of the stability of performance and of the rat's mastery of the experimental paradigm. These trials also provide fixed anchors to which the rat can compare the variable payoff offered on the middle trial of each triad.

Sandwiched between the leading and trailing trials was a “test” trial on which the price and pulse frequency were drawn without replacement from the randomized list. Trial triads were run repeatedly until all elements in the list had been tested. A complete set of such triads is called a “survey” of the mountain. Four of the rats were able to complete a survey during a single experimental session (maximum duration: 9 h) in at least one of the probability conditions; in the remaining cases, multiple sessions were required to work through the entire list of test-trial parameters constituting a survey. Data from the test trials were used to fit the Reward-Mountain model and to test its predictions.

Reward delivery was certain upon satisfaction of the 1 s response requirement on leading and trailing trials. On test trials, reward delivery was either certain (*Pr* = 1.00) or probabilistic (*Pr* = 0.75 or *Pr* = 0.50). Only one lever was armed on any given trial.

For rat MA5, the same lever served as manipulandum for both certain (*Pr* = 1.00) and risky (*Pr* < 1.00). For rats DE01, DE03, DE07, DE14, DE15, DE16, DE19, DE20, and PD8, one lever was mapped to all trials in which reward was certain and the other lever was mapped to all trials in which reward was risky. In all cases, a steady cue light mounted above the lever signaled that reward would be delivered with certainty, while a flashing cue light (300 ms on, 300 ms off) signaled that reward would be delivered probabilistically.

Performance was deemed stable when, by visual inspection, time-allocation ratios were reliably near maximal on high-payoff trials, near minimal on low-payoff trials, and intermediate on moderate-payoff trials. After stable performance had been observed throughout 8 consecutive surveys of the reward mountain, the probability of reinforcement was changed to 0.50 (P1vp5). A new master list was created by amalgamating the frequency, price, and radial pseudo-sweeps for the certain (*Pr* = 1.00) condition with the pseudo-sweeps for new risky (*Pr* = 0.50) condition. As above, the central 5 points of each pseudo-sweep were double-sampled. The list was presented again, in triads for which the 2nd trial was now randomly drawn without replacement from the new master list.

When performance on this new probability condition was judged stable by visual inspection for 8 consecutive surveys, the location of the levers providing certain (*Pr* = 1.00) and risky (*Pr* = 0.50) rewards was switched (P1vp5sw). A steady cue light still signaled that the lever would always deliver reward, and a flashing cue light still signaled that the lever would not always deliver reward, but the mapping of levers to those probabilities was inverted. If, for example, the lever delivering certain rewards was on the left side for the previous two probability conditions, the right lever would now fulfill that role, and vice-versa. This switch enabled us to partly control for side-preferences.

After rats completed 8 stable surveys comparing certain and risky rewards, the probability was changed again to 0.75 (P1vp75sw). A master list was constructed again by amalgamating pseudo-sweeps for the *Pr* = 1.00 condition with those for the *Pr* = 0.75 condition, double-sampling the central 5 price-frequency pairs as above. The levers maintained their inverse mapping, and the 2nd trial of every triad was drawn at random without replacement from this final master list. Data collection continued until 8 stable surveys were collected under this switched certain (*Pr* = 1.00) compared to risky (*Pr* = 0.75) condition.

Rats DE01, DE03, and DE07 began the experiment as rat MA5, with probabilities mapped to the same lever but signaled with a steady or flashing light. As no difference in performance was observed under either P1vp75 or P1vp5 between certain and risky conditions, mapping of levers to probabilities was instituted, as described above. Then, 8 stable surveys were obtained at P1vp75 and 8 (rat DE01), 5 (rat DE03) or 6 (rat DE07) surveys at P1vp5.

In summary, rats were presented with a triad sequence of trials in which the first delivered strong, inexpensive stimulation, the second drew price, frequency, and risk values from the P1vp75, P1vp5, P1vp5sw, or P1vp75sw lists, and a third trial delivered weak, inexpensive stimulation. The order of the probability conditions was always P1vp75, followed by P1vp5, P1vp5sw, and finally P1vp75sw. Rat MA5 did not undergo the lever-switch conditions, as a single lever was used for both conditions. Due to the substantial duration of the individual conditions, most rats did not complete the entire experiment and fell ill prior to completion of all conditions. Rats DE01, DE7, DE14, DE15, DE16, and PD8 made it at least part-way through conditions P1vp75, P1vp5, P1vp5sw, P1vp5, P1vp75, and P1vp5, respectively.

### 2.3. Statistical analysis

The dependent measure was corrected time allocation, the proportion of trial time the animal spent working for brain stimulation rewards. The correction was twofold. First, lever releases lasting less than 1 s were included along with lever holds in our measure of corrected work time (Hernandez et al., 2010; Breton et al., 2013); during such brief releases, the rat typically remains at the lever with its paw held upon it or just above it. Second, lever releases and holds prior to the receipt of the first reward were excluded from the calculation of time allocation. This was done because on test trials, the values of the independent variables were selected randomly, and the rat had to have earned the first reward in order to discover its price and intensity. Corrected time allocation was therefore calculated as the total amount of time the lever was depressed (for any period of time) or released for less than 1 s, divided by the total trial time, during the portion of the trial following receipt of the first reward. If no rewards were earned, time allocation was assigned a value of zero.

The Reward-Mountain Model surface (Equation 1; Breton et al., 2013) was fit to the corrected time allocation on test trials, measured at each combination of frequency (*F*), price (*P*), and probability condition. The values of the *F*_*hm*_ and *P*_*e*_ parameters were obtained by back-solving equations 2 and 3, with *FF* = *FF*_*hm*_ and *SPr* = *SP*_*e*_, respectively. Values for *F*_*NearMax*_ (the pulse frequency inducing a half-maximal spike rate in putative reward neurons) and *F*_*bend*_ (the parameter governing the abruptness of the transition between the rising and asymptotic segments of the function) (Breton et al., 2013) were fixed to the means of those reported by Solomon et al. (2010): *F*_*NearMax*_: 338.8 pulses per second; *F*_*bend*_: 20.42 pulses per second. Values for *SP*_*min*_ (the minimum subjective price) and *SP*_*bend*_ (the parameter governing the sharpness of the bend in the subjective-price function) (Breton et al., 2013) were fixed to to the means of those reported by Solomon et al. (2007): *SP*_*min*_: 1.75 s; *SP*_*bend*_: 0.57.

(1) $$TA=\text{​ }(\text{​}TA_{max}-\;TA_{min}\text{​})\text{​}\frac{{\left(\frac{FF^{G}}{F^{G}+FF_{hm}^{G}}\right)}^{A}}{{\left(\frac{FF^{G}}{FF^{G}+F_{hm}^{G}}\right)}^{A}+{\left(\frac{SP}{SP_{e}}\right)}^{A}}\text{​ }+\text{ ​}TA_{min}$$

where

(2) $$\begin{array}{l} FF=F_{bend}\;\times \;[Ln\;\left(1+e^{\frac{F_{NearMax}}{F_{bend}}}\right)\\ \;-Ln\;\left(1+e^{\frac{F_{NearMax-F}}{F_{bend}}}\right)] \end{array}$$

(3) $$SPr=SP_{min}+SP_{bend}\times \;Ln\;\left(1+e^{\frac{\left(P-SP_{min}\right)}{SP_{bend}}}\right)$$

(4) $$SP_{e}=\frac{RI_{max}\times Pr}{U_{e}\;\times \;(1+\xi )}$$

The symbols in the above equations are defined in Table 1.

**Table 1:** **Definition of symbols and acronyms**.

*A*	Price-sensitivity exponent
*BSR*	Brain stimulation reward
*F*	Pulse frequency
*FF*	Firing frequency induced by *F*
*F*_*bend*_	Shape parameter of the frequency-response function
*FF*_*hm*_	Firing frequency that produces a reward of half-maximal intensity
*FF*_*NearMax*_	Position parameter of the frequency-response function
*G*	Reward-growth exponent
*P*	Price (required cumulative hold time)
*Pr*	Subjective reward probability
*RI*	Subjective reward intensity
*RI*_*max*_	Maximum subjective reward intensity
*SP*	Subjective price (opportunity cost)
*SP*_*bend*_	Shape parameter of the subjective price function
*SP*_*e*_	Subjective price at which the payoff from a maximal reward equals the payoff from alternate activities
*SP*_*min*_	Minimum subjective price
*TA*	Time allocation to working for reward
*TA*_*max*_	Maximum time allocation
*TA*_*min*_	Minimum time allocation
ξ	Parameter of subjective effort-cost function

The only parameters of the model that were free to vary between probability conditions were *F*_*hm*_, the location of the surface along the frequency axis, and *P*_*e*_, its location along the price axis. Slope (*A*, *G*), ceiling (*TA*_*max*_) and floor (*TA*_*min*_) parameters were not free to vary between probability conditions. Separate fits were conducted for P1vp75, P1vp5, P1vp5sw, and P1vp75sw conditions.

A bootstrapping approach was used to derive the confidence intervals around *F*_*hm*_, *P*_*e*_, and the differences between the estimates obtained when reward delivery was either certain or probabilistic. The bootstrapping and fitting algorithms were both implemented in MATLAB R2013b (The Mathworks, Natick, MA). Corrected time allocation values were sampled 1000 times from the observed data with replacement. For example, if 8 time allocation values were observed at a particular price, frequency, and reward probability, the bootstrapping procedure would obtain 1000 samples of 8 time allocation values obtained pseudo-randomly from that list of 8 (with replacement). A mountain surface was fit to each of the 1000 re-sampled replications, thereby producing 1000 estimates of *F*_*hm*_ and *P*_*e*_ for each probability condition. The 95%, bootstrap-derived confidence interval about *F*_*hm*_ and *P*_*e*_ was defined as the range within which the central 950 *F*_*hm*_ and *P*_*e*_ values lay (i.e., all values excluding the lowest and highest 25). Similarly, we computed the difference between estimates of *F*_*hm*_ and *P*_*e*_ during riskless and risky trials by obtaining the difference for each replication. In other words, each replication had an estimate of *F*_*hm*_ for riskless (*Pr* = 1.00) trials and one for risky (*Pr* = 0.75 or *Pr* = 0.50) trials, and the parameter difference in *F*_*hm*_ for the replication was the difference between each. The 95% bootstrap-derived confidence interval about the difference in *F*_*hm*_ and *P*_*e*_ was defined as the range within which the central 950 sample differences lay for each parameter. Our criterion for statistical reliability was non-overlap of the 95% confidence interval about the difference with 0. A probability-induced difference in *F*_*hm*_ or *P*_*e*_ was therefore considered statistically reliable if and only if the 95% confidence interval about the difference did not include 0.

Graphical representations of the results, including figures for publication, were generated using MATLAB R2013B.

## 3. Results

### 3.1. Fit of the mountain structure

**Figure 2:**
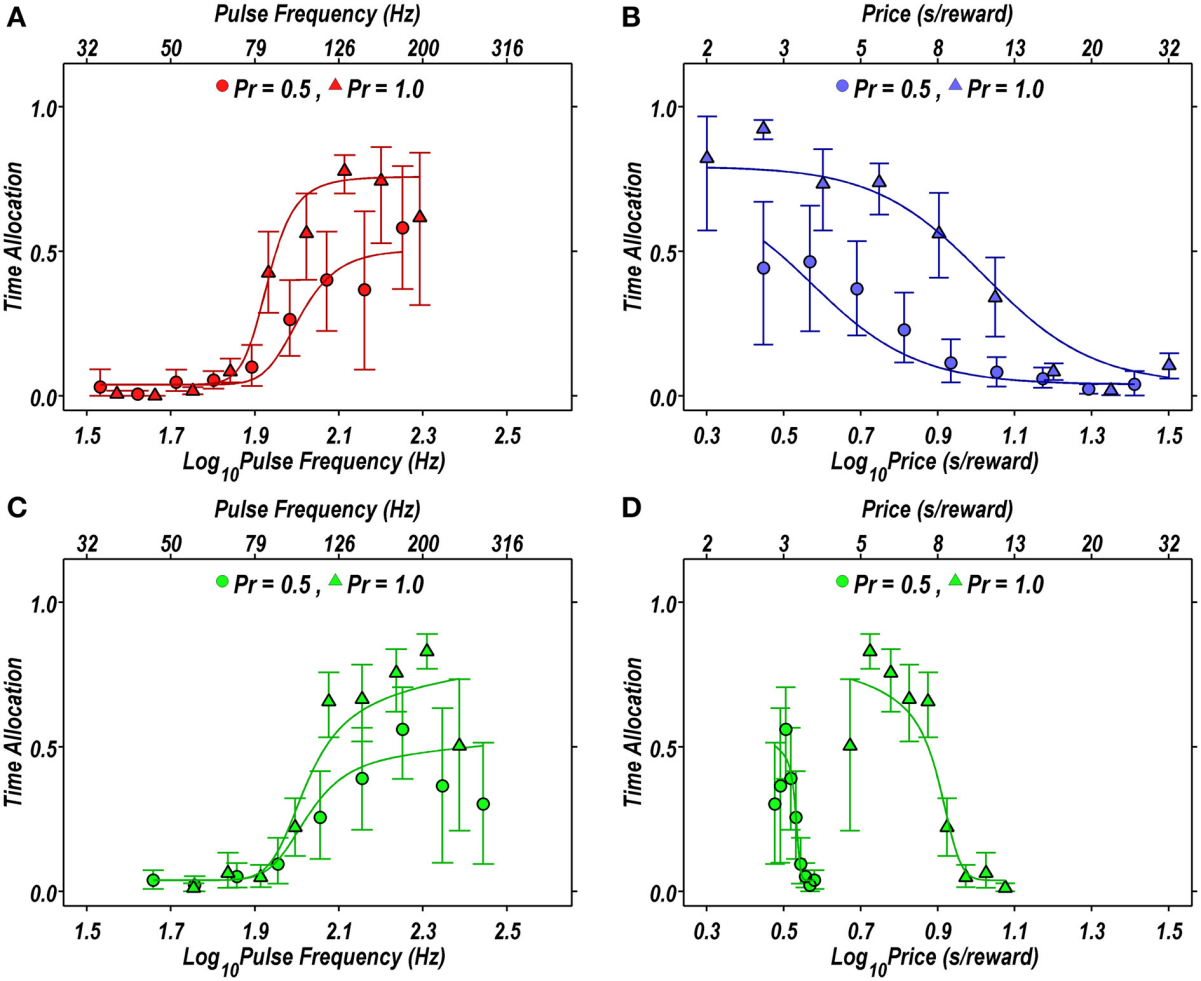
**Projections of the reward mountain model fit to the data from a single subject (rat DE03) in the P1vp5 condition, projected in two dimensions. (A–D)** Proportion of time allocated to self-stimulation when the probability of reward delivery is 1.00 (triangles) or 0.50 (circles) compared to the proportion of time predicted by the fit of the Reward-Mountain model (solid lines). In **(B)**, risk induces a leftward shift in the rat's willingness to lever-press. **(C,D)** Projection of the radial pseudo-sweep on the pulse frequency **(C)** and price **(D)** axes. In **(A,C)**, the rightward shift in the response-strength curve induced by risk results from the displacement of the reward mountain along a dimension that does not appear in the 2D representation.

Figure 2 illustrates the fit of the Reward-Mountain model, as viewed from the 2D perspective. The fitted models approximate the rat's performance quite closely for both risky (circles) and riskless (triangles) rewards.

**Figure 3:**
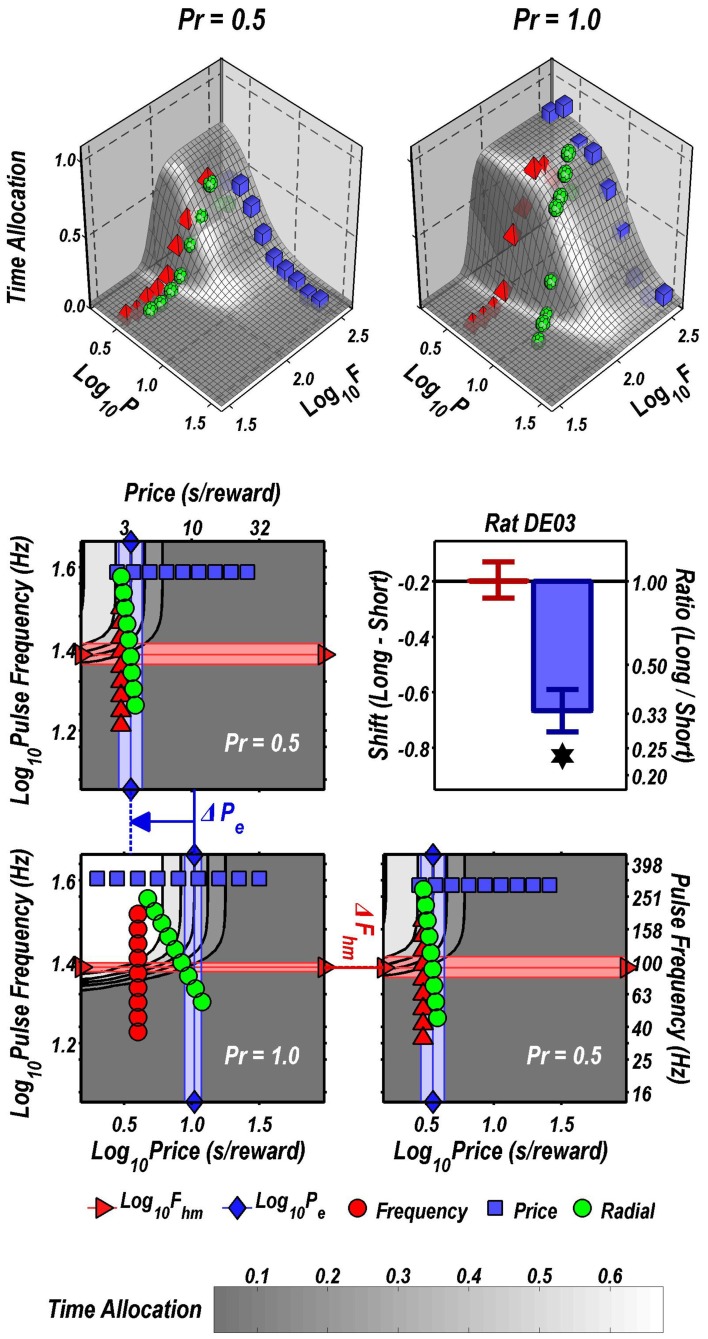
**3D depictions of the data plotted in 2D in Figure 2. Upper row:** fitted surfaces and pseudo-sweep data. **Middle and bottom row:** contour maps showing shifts in the location parameters produced by reducing reward probability from 1.00 to 0.5 in rat DE03. The shaded bands are the 95% confidence intervals about the location-parameter estimates. The contour graph of the data obtained at *Pr* = 0.5 is shown in the lower left. For ease of comparison along both dimensions, the contour graph of the data obtained at *Pr* = 1.00 is shown twice, once above and once to the right of the contour graph of the data obtained at *Pr* = 0.50. The decrease in reward probability has shifted the reward mountain leftward along the price axis. In contrast, the position of the mountain along the pulse frequency axis has not changed. The magnitude of the shifts is summarized in the bargraph.

The data in Figure 2 are replotted in 3D in Figure 3. The upper row shows the fitted surfaces as well as the spatial relationship between the three pseudo-sweeps. The lower row summarizes the 3D representations in contour-map format.

The contour maps are replotted in Figure 3 to reveal the direction in which the mountain has been shifted by the change in reward probability. As is evident from a comparison of the two lower panels, there is a negligible shift along the pulse-frequency axis and near-total overlap of the pink 95% confidence intervals around the location parameter (*F*_*hm*_) estimates for the two mountains. In contrast, the shift along the price axis is large and reliable, as is evident from the displacement between the light-blue 95% confidence intervals around the location parameter (*P*_*e*_) estimates in the two contour graphs on the left. Delivering the reward with 0.5 probability reduced the prices that the rat was willing to pay threefold, as indicated by the change in the *P*_*e*_ parameter.

Although there appears to be displacement along both axes in the 2D view (Figure 2), the 3D depictions (Figure 3) show that the reward mountain moved only along the price axis. The apparent displacement in Figures 2A,C arises from the diagonal orientation of the fitted surface where it intersects the frequency and radial pseudo-sweeps (Figure 3, top row) (Hernandez et al., 2010).

### 3.2. Stability of *F*_*hm*_ and *P*_*e*_

The *Pr* = 1.00 condition is common to all phases of the experiment. In the absence of any drift, the estimated *F*_*hm*_ and *P*_*e*_ values for rewards delivered with certainty would be identical in all phases, thereby justifying a single, three-way comparison between *Pr* = 1.00, *Pr* = 0.75, and *Pr* = 0.50 trials across all four phases. However, the duration of the experiment was very substantial, 4.9 months after the completion of training, on average. We tested the null hypothesis that the reward mountain was stable over these long time periods by comparing the location-parameter estimates obtained in the multiple tests carried out with *Pr* = 1.00.

**Figure 4:**
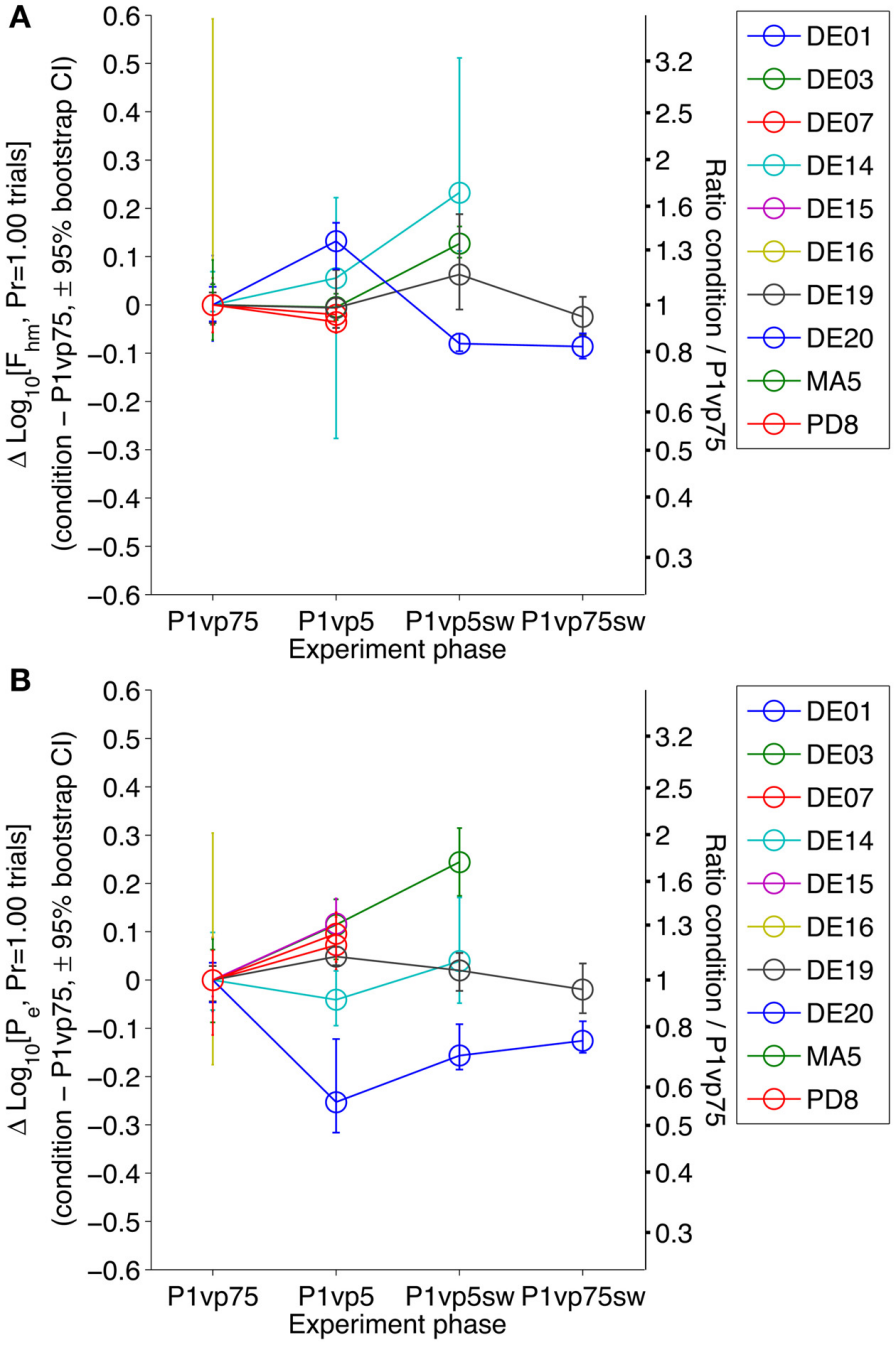
**Stability of *F*_*hm*_ and *P*_*e*_ in the riskless condition across experimental phases**. The estimated *F*_*hm*_ **(A)** and *P*_*e*_ **(B)** parameters estimated from the results obtained in the *Pr* = 1.00 condition are compared across each phase of the experiment, normalized to the values obtained in the first condition (P1vp75). There are statistically reliable (although non-systematic) changes from one condition to the next in all subjects.

Figure 4 shows the difference between the estimates of the *F*_*hm*_ (top) and *P*_*e*_ (bottom) parameters obtained in the first phase of the experiment and in each subsequent phase, along with the associated bootstrap-derived confidence intervals. In every subject, there were statistically reliable changes in the location parameters (confidence intervals about the difference that do not include 0) at one or more points in time. These results make it unreasonable to assume that the subject and electrode/brain interface remained absolutely stable over the many months of testing. Since probabilistic and risk-less trials were presented in a randomly inter-digitated fashion, these drifts in *F*_*hm*_ and *P*_*e*_ would constitute part of the statistical noise were a single analysis conducted across all conditions. Instead, we carried out separate comparisons between the data from the riskless and risky conditions at each phase of the experiment.

### 3.3. Effects of probability on *F*_*hm*_ and *P*_*e*_

**Figure 5:**
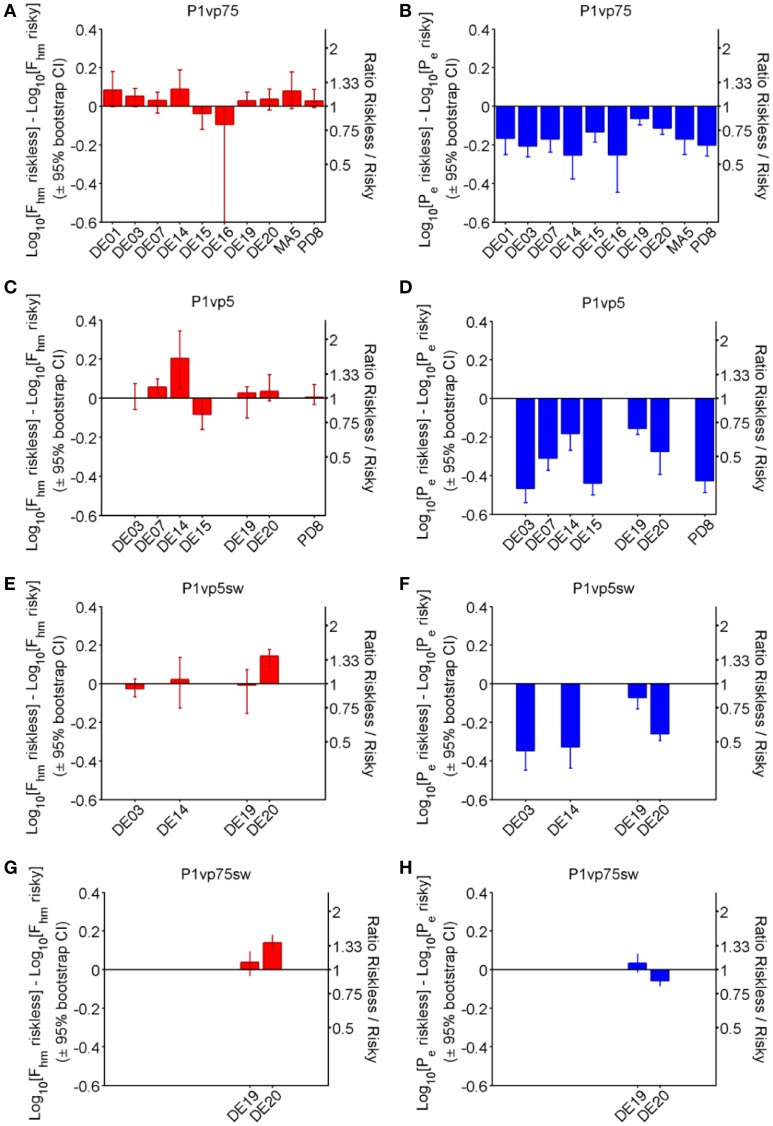
**Shift in location-parameter estimates for each condition**. Bar graphs provide the magnitude (± 95% bootstrap confidence interval) of the difference in *F*_*hm*_ **(A,C,E,G)** and *P*_*e*_ **(B,D,F,H)** from riskless (*Pr* = 1.00) to risky (*Pr* < 1) conditions in each phase of the experiment. Positive numbers indicate that the estimate for the risky conditions is greater, whereas negative numbers indicate that the estimate for the risky conditions is smaller.

Figure 5 shows the effect of varying reward probability on the location-parameter estimates for each subject and experimental phase. The bar graphs in the left-hand panels (red: A, C, E, G) depict the estimated differences in *F*_*hm*_ along with the associated bootstrap-estimated 95% confidence interval, whereas the right-hand panels (blue: B, D, F, H) depict the corresponding differences in the *P*_*e*_ estimates. Whereas differences in *F*_*hm*_ produced by decreasing reward probability were typically small, inconsistent across experimental phases, and variable across rats, *P*_*e*_ estimates decreased in a probability-dependent manner that was largely consistent across experimental phases and subjects.

Although 4 animals (rats DE14, DE15, DE16, DE19) showed a statistically reliable shift in *F*_*hm*_ when reward probability was initially decreased from 1.00 to 0.75 (panel A), the median difference across all animals is small (0.033 common logarithmic units, a 1.08-fold change) and inconsistent (interquartile range: 0.026–0.079 common logarithmic units). In contrast, all rats showed reliable reductions in *P*_*e*_ as a result of the decrease in reward probability (panel B). The median difference between the *P*_*e*_ estimates for the riskless and risky rewards is 0.170 common logarithmic units, a (1.48-fold) decrease, with an interquartile range of 0.134–0.207 common logarithmic units (1.36-fold to 1.61-fold).

The further reduction of reward probability to 0.50 (Panel C) altered the *F*_*hm*_ estimates systematically in only two of the seven rats tested. The median value of this parameter increased by only 0.028 common logarithmic units (a 1.07-fold change), with an interquartile range of 0.0015–0.051 common logarithmic units. In contrast, there was a large and consistent decrease in *P*_*e*_ in all rats. The median shift in *P*_*e*_ was 0.311 common logarithmic units (a 2.04-fold change), and the interquartile range was −0.436 to −0.206 common logarithmic units.

Only four rats remained in the third phase of the experiment, when the lever mapping was reversed and *Pr* = 0.5 was again compared to *Pr* = 1.00. The manipulation of reward probability shifted the *F*_*hm*_ estimate in only one of these subjects (rat DE20), whereas no reliable change had been observed with this rat with the original mapping between lever and risk. The median shift in *F*_*hm*_ in this phase of the experiment was 0.0075 common logarithmic units (1.02-fold), with an interquartile range of −0.0171 to 0.0835 common logarithmic units. In contrast, large, reliable decreases in *P*_*e*_ were obtained in three of the four rats, with a median value of 0.294 common logarithmic units (1.97-fold) and an interquartile range of −0.339 to −0.166 common logarithmic units.

Only two rats (DE19, DE20) completed the final phase of the experiment, which entailed comparison of *Pr* = 0.75 to *Pr* = 1.00 with the new mapping between lever and risk in effect. With the original lever mapping in effect, the reduction in probability had produced a small, marginally reliable shift in *F*_*hm*_ in rat DE19, but with the new lever mapping, there was no longer a reliable change in the *F*_*hm*_ estimate. Rat DE20 did show an increase in *F*_*hm*_ with the new lever mapping but had not shown one with the original mapping. Both rats had shown decreases in *P*_*e*_ following reduction of the reward probability to 0.75 with the original mapping; such an effect was seen again with the new lever mapping in rat DE20 but not in rat DE19.

### 3.4. The effect of decreasing reward probability, collapsed across lever mappings

**Figure 6:**
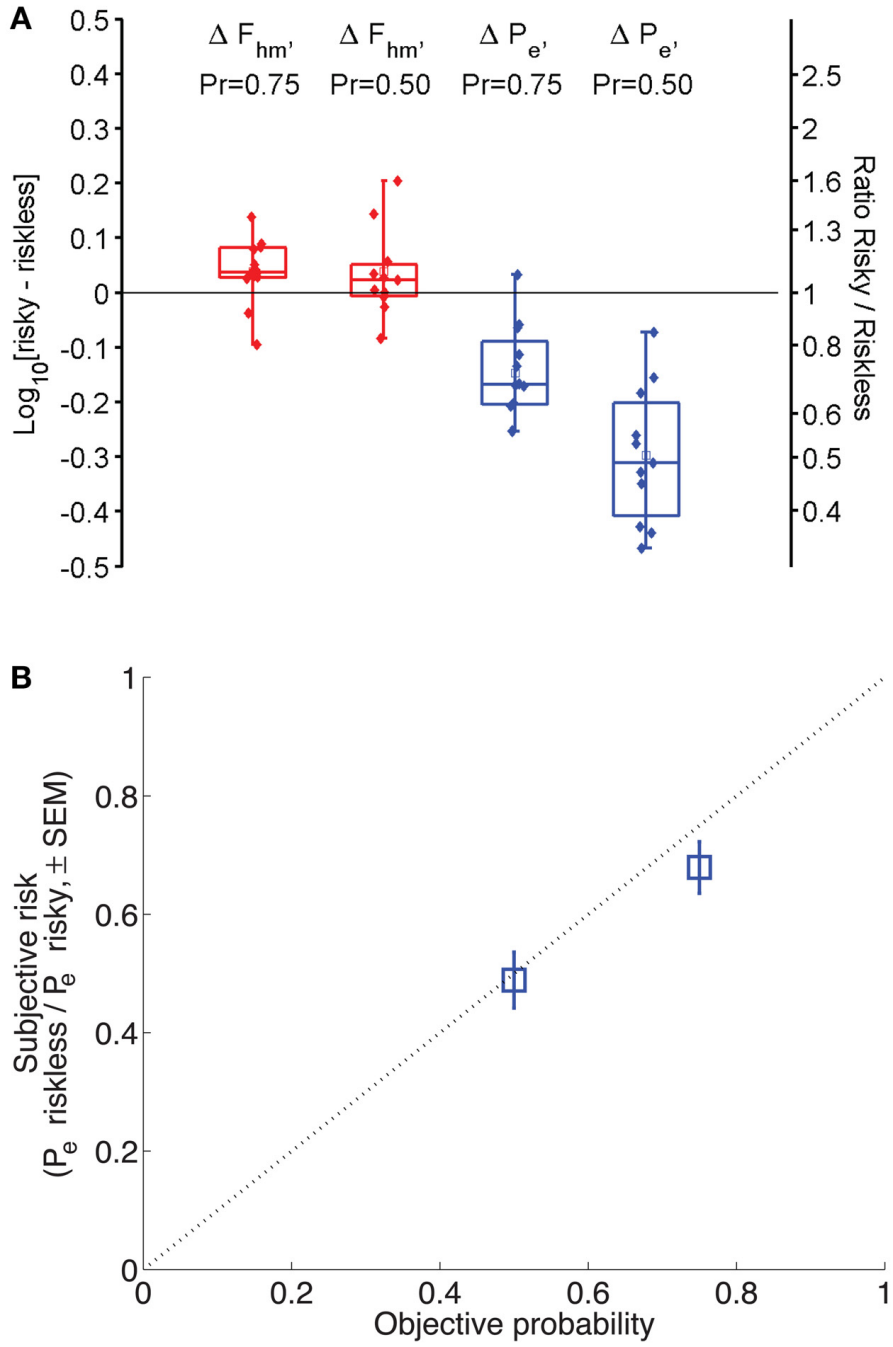
**(A)** Magnitude of the shift in *F*_*hm*_ and *P*_*e*_ across all conditions. Box-whisker plots show the change in *F*_*hm*_ and *P*_*e*_ resulting from a decrease in reward probability to 0.75 or 0.50. Squares represent means, whiskers represent the full range of differences. Negative numbers indicate that risk decreases the estimate; positive numbers indicate that risk increases it. Filled circles represent differences for each animal in each condition. **(B)** Derived subjective-to-objective mapping of probability. The dotted line represents the case in which subjective and objective probabilities are equal. If subjective and objective probabilities are equal when *Pr* = 1.00, the anti-log of the change in Log_10_[*P*_*e*_] is an index of the subjective probability in the risky condition. Blue squares indicate the mean subjective reward probabilities derived in this fashion (± s.e.m.).

The shift in *F*_*hm*_ and *P*_*e*_ induced by a given reduction in reward probability was collapsed across animals and lever-mapping conditions to provide a global estimate of how probability affects each parameter. Panel A of Figure 6 shows a box-whisker plot of the shifts in *F*_*hm*_ and *P*_*e*_ observed by reducing reward probability from 1.00 to 0.75 and from 1.00 to 0.50 (combining the data from the P1vp75 and P1vp75sw conditions and from the P1vp5 and P1vp5sw conditions). The shift in *F*_*hm*_ is close to 0 for both reductions in reward probability. The median change in *F*_*hm*_ for *Pr* = 0.75 is an increase of only 0.037 common logarithmic units (1.09-fold), with an interquartile range of 0.028 to 0.0817 common logarithmic units, whereas the median change for *Pr* = 0.50 is an increase of only 0.023 common logarithmic units (1.05-fold) with an interquartile range of −0.006 to 0.051 common logarithmic units. In contrast, *P*_*e*_ is shifted systematically as reward probability is decreased, an effect that grows in magnitude with the change in reward probability. The median change in *P*_*e*_ for *Pr* = 0.75 is a decrease of 0.168 common logarithmic units (1.47-fold), with an interquartile range of 0.089 to 0.2041 common logarithmic units, whereas the median change for *Pr* = 0.50 is a decrease of 0.311 common logarithmic units (2.05-fold) with an interquartile range of 0.202 to 0.408 common logarithmic units.

## 4. Discussion

### 4.1. Utility of the mountain model: implications of the present findings

Studies of the neural basis of reward-seeking have employed a wide array of manipulations, including perturbation of physiological regulatory systems, drugs, lesions, and optogenetic interventions. The Reward-Mountain Model has been used to infer the stage of neural processing at which such manipulations act to alter reward seeking. According to the model, manipulations that alter the pulse frequency that produces a half-maximal reward (*F*_*hm*_) act prior to the output of a circuit that integrates the aggregate activity induced by the stimulation electrode. In other words, manipulations that alter *F*_*hm*_ are presumed to operate on neurons responsible for the rewarding effect of the electrical stimulation. In contrast, manipulations that alter the price that drives equipreference between a maximally rewarding train of brain stimulation and all other activities (*P*_*e*_) occur at or beyond the output of the integration network, modifying the payoff from self-stimulation activities, rescaling the output of the peak detector, or changing the payoff from alternate activities. Thus, manipulations that alter *P*_*e*_ produce their effects not by acting on the primary neurons responsible for brain stimulation reward (depicted to the left of the vertical line in Figure 1), but rather at some later stage instead.

Previous tests of the validity of the Reward-Mountain Model have focussed on manipulations known to affect the directly stimulated neurons subserving BSR or the circuitry that integrates their output and translates it into a subjective reward intensity. For example, Arvanitogiannis and Shizgal (2008) tested the predictions of the Reward-Mountain Model concerning alterations in stimulation current and train duration, both of which are posited by the model to act prior to the output of the peak-detection stage (to the left of the vertical line in Figure 1). More recently, Breton et al. (2013) revisited the effects of varying the train duration, using the same updated experimental procedure as that employed in the present paper. Other studies have applied the Reward-Mountain Model to infer the stage of processing at which cocaine (Hernandez et al., 2010), the specific dopamine transporter blocker, GBR-12909 (Hernandez et al., 2012), the dopamine antagonist, pimozide (Trujillo-Pisanty et al., 2014), and the cannabinoid antagonist, AM-251 (Trujillo-Pisanty et al., 2011), act to alter performance for rewarding electrical brain stimulation. The predominant effect of these pharmacological interventions has been to alter *P*_*e*_, sometimes with small, inconsistent effects on *F*_*hm*_. These findings position the actions of the drugs to the right of the vertical line in Figure 1, beyond the point where the peak reward-intensity signal is generated. However, no validation study has been carried out until now to verify that the Reward-Mountain Model indeed isolates such effects.

We report a large, probability-dependent decrease in *P*_*e*_ that is accompanied, in some cases, by small, unreliable, and inconsistent shifts in *F*_*hm*_. Overall, these results support the predictions of the Reward-Mountain Model. They show that the model is indeed capable of correctly determining the stage of processing affected by a manipulation that alters payoff without changing the neural activity directly induced by the rewarding stimulation. Thus, the findings complement those reported by Breton et al. (2013) and Arvanitogiannis and Shizgal (2008) Together, the three studies confirm multiple predictions of the Reward-Mountain Model and provide a firm empirical basis for it.

The present findings support further application of the Reward-Mountain Model in studies of the neural basis of reward seeking. For example, the model could be used to test hypotheses concerning the identity of the directly stimulated neurons underlying brain stimulation reward. Such a hypothesis predicts that lesioning or optogenetic silencing of the neurons in question would increase *F*_*hm*_ without altering *P*_*e*_. Similarly, a pharmacological, pharmacogenetic, or optogenetic intervention that boosted signaling in the directly activated substrate would decrease *F*_*hm*_ without altering *P*_*e*_. Manipulations that decrease the subjective effort cost of lever-pressing would increase *P*_*e*_ without altering *F*_*hm*_, and those that scale down the magnitude of all rewards by a constant factor (Hernandez et al., 2010) would decrease *P*_*e*_ without altering *F*_*hm*_. No previously proposed method can make such distinctions.

The application of the Reward-Mountain Model is limited neither to electrical rewards nor to lever-pressing. It should be feasible to perform a similar analysis using a natural reward, such as sucrose, or a different operant response, such as nose-poking or wheel turning. An analogous formulation would apply: the objective strength of the reward (e.g., the concentration of sucrose) and its opportunity cost would each be mapped psychophysically to subjective determinants of choice; the results would then be combined in scalar fashion and compared to the payoff from alternate activities to determine time allocation. A 3D “sucrose mountain,” would be fit to data obtained by varying sucrose concentration and work requirements. In this way, one could determine the stage(s) of processing at which changes in physiological state or manipulations of neural signaling alter sucrose seeking.

### 4.2. Implications for theories of rodent decision making under uncertainty

Three levels of reward probability were tested in this study: 0.50, 0.75, and 1.00. According to the Reward-Mountain Model, the position of the 3D structure along the price axis depends on the corresponding *subjective* probabilities. Thus, the results provide information about the psychophysical transformation of objective probabilities into subjective ones. Over the tested range, the estimated subjective probabilities correspond generally to the objective values.

The estimates of subjective probability are derived from the shifts of the 3D structure along the price axis as reward probability was decreased from 1.00 to either 0.75 or 0.50. The reasoning is as follows: According to the Reward-Mountain Model, changes in subjective price compensate for changes in subjective reward probability. To hold payoff constant for a reward of a given intensity, its subjective price would have to be halved in order to compensate for a decrease in subjective reward probability from 1.00 to 0.50. Measurement of the function relating objective and subjective prices indicates that the two are essentially equal over the range spanned by our *P*_*e*_ estimates (Solomon et al., 2007). If so, the change in *P*_*e*_ is equal to the change in subjective probability.

Panel A of Figure 6 shows that reduction of the objective probability of reward delivery from 1.00 to 0.50 decreased the median *P*_*e*_ estimate by 0.311 common logarithmic units. The antilog of −0.311 is 0.49, which, as explained above, corresponds to the estimated *subjective* probability. The diagonal line in Panel B of Figure 6 represents the case in which subjective and objective probabilities are equal. Note that the error bar (representing the standard error of the mean) around the subjective-probability estimate for *Pr* = 0.50 overlaps this diagonal and that the error bar around the subjective-probability estimate for *Pr* = 0.75 does not miss by much. Thus, there is little evidence that subjective-probability estimates deviate from the objective values.

In studies of human decision making under uncertainty, extreme probabilities are overweighted when the participants learn the nature of the prospects from verbal descriptions (Kahneman and Tversky, 1979; Hau et al., 2008). However, when human participants learn about probabilities from experience, as non-human animals must, extreme probabilities are underweighted instead (Hau et al., 2008). Neither distortion was likely to apply in the present study, which did not expose the subjects to rare events and did not entail choices between pairs of experimenter-controlled rewards presented simultaneously. Although the risky option was variable and stochastic, it was predictably so: The mapping between lever and reward probability remained stable for many weeks at a time. Under these conditions, the rats appear to have arrived at accurate subjective assessments of reward probability and to have used these in a consistent fashion.

### 4.3. Future directions

Dramatic recent advances in neuroscientific methods (e.g., Yizhar et al., 2011; Chung and Deisseroth, 2013; Deisseroth, 2014) have led to rapid progress in tracing and visualizing neural circuitry related to the evaluation and pursuit of rewards. For these exciting new approaches to realize their full potential, they must be combined with behavioral methods of sufficient power, precision, and discrimination. The Reward Mountain Model and the measurement methods derived from it were developed to address such challenges. The results of this experiment support a key postulate of the model and thus provide encouragement for future applications. Substituting optogenetic methods for the less specific, electrical stimulation employed in the present study should strengthen the potential of this approach to link well-quantified psychological processes involved in reward seeking with the activity of specific neural pathways.

## Author contributions

Yannick-André Breton carried out the experiments. In addition, he participated in planning the experiments, carrying out the statistical analysis, interpreting the results and writing the manuscript. Kent Conover developed the statistical approach and the data analysis software. In addition, he participated in carrying out the statistical analysis and revising the manuscript. Peter Shizgal participated in planning the experiments, interpreting the results and writing the manuscript.

## Funding

Funding was provided by a Bourse de Doctorat de Recherche scholarship from the Fonds Québécois de Recherche sur la Nature et les Technologies to Yannick-André Breton, grants from the Canadian Institutes of Health Research (Peter Shizgal, p.i., MOP74577) and the “Fonds de recherche du Québec - Santé” (Shimon Amir, p.i.), and support to Peter Shizgal from the Concordia University Research Chairs program.

### Conflict of interest statement

The authors declare that the research was conducted in the absence of any commercial or financial relationships that could be construed as a potential conflict of interest.
